# Metagenomics-Based Discovery of Malachite Green-Degradation Gene Families and Enzymes From Mangrove Sediment

**DOI:** 10.3389/fmicb.2018.02187

**Published:** 2018-09-11

**Authors:** Wu Qu, Tan Liu, Dexiang Wang, Guolin Hong, Jing Zhao

**Affiliations:** ^1^School of Life Sciences, Xiamen University, Xiamen, China; ^2^College of Ocean and Earth Sciences, Xiamen University, Xiamen, China; ^3^The Department of Laboratory Medicine, The First Affiliated Hospital of Xiamen University, Xiamen, China

**Keywords:** mangrove sediment, metagenome, MG biodegradation, gene expression, biochemical analysis, degradation pathway

## Abstract

Malachite green (MG) is an organic contaminant and the effluents with MG negatively influence the health and balance of the coastal and marine ecosystem. The diverse and abundant microbial communities inhabiting in mangroves participate actively in various ecological processes. Metagenomic sequencing from mangrove sediments was applied to excavate the resources MG-degradation genes (MDGs) and to assess the potential of their corresponding enzymes. A data set of 10 GB was assembled into 33,756 contigs and 44,743 ORFs were predicted. In the data set, 666 bacterial genera and 13 pollutant degradation pathways were found. *Proteobacteria* and *Actinobacteria* were the most dominate phyla in taxonomic assignment. A total of 44 putative MDGs were revealed and possibly derived from 30 bacterial genera, most of which belonged to the phyla of *Proteobacteria* and *Bacteroidetes*. The MDGs belonged to three gene families, including *peroxidase* genes (up to 93.54% of total MDGs), *laccase* (3.40%), and *p450* (3.06%). Of the three gene families, three representatives (Mgv-rLACC, Mgv-rPOD, and Mgv-rCYP) which had lower similarities to the closest sequences in GenBank were prokaryotic expressed and their enzymes were characterized. Three recombinant proteins showed different MG-degrading activities. Mgv-rPOD had the strongest activity which decolorized 97.3% of MG (300 mg/L) within 40 min. In addition, Mgv-rPOD showed a more complete process of MG degradation compared with other two recombinant proteins according to the intermediates detected by LC-MS. Furthermore, the high MG-degrading activity was maintained at low temperature (20°C), wider pH range, and the existence of metal ions and chelating agent. Mgv-rLACC and Mgv-rCYP also removed 63.7% and 54.1% of MG (20 mg/L) within 24 h, respectively. The results could provide a broad insight into discovering abundant genetic resources and an effective strategy to access the eco-friendly way for preventing coastal pollution.

## Introduction

The application of synthetic dyes is extensive in the fields of textile, pharmaceutical, cosmetics, paper making, solar cells, and acrylic industries (Chang and Lin, 2001). Due to various risks on environment and human health, strategies for the removal of dyes have attracted increasing attention from health professionals and environmentalists (Srivastava et al., 2004; Venil and Lakshmanaperumalsamy, 2010; Gopinathan et al., 2015). Malachite green (MG), in particular, is one typical of synthetic dyes and is widely used in dyeing of silk, leather, and paper, which has been shown to harm humans and animals because of toxicity, mutagenicity, and carcinogenicity (Srivastava et al., 2004; Gopinathan et al., 2015). MG is a triphenylmethane dye and highly soluble in water (Yong et al., 2015). MG can be metabolized into leucomalachite green (LMG) (Yong et al., 2015) and remains in fish muscles, fat, and organs with a half-life of about 10 days and even longer (Chen and Miao, 2010). MG is also persistent in the environment and its half-life in sediment can range from 12.9 to 50.34 days (Jiang, 2011). Despite the global ban on MG in aquaculture, 1–15% of dyes are estimated being discharged within effluents, which seriously inhibit survival, development, and reproductive of aquatic organisms (Chang et al., 2016). The removal or reduction of MG can be processed by chemicals methods or biological treatments. Chemicals methods, such as photodegradation (Algubury, 2016; Pathania et al., 2016; Liang et al., 2017), have some disadvantages of high costs and secondary pollutant generation (Tayabali et al., 2017). Bioremediation strategy may constitute an alternative approach to conventional physicochemical methods, benefiting from the potential of indigenous microorganisms to metabolize anthropogenic compounds (Grosser et al., 1991; Tayabali et al., 2017).

Mangrove ecosystems constitute 60–70% of the coastline in the tropical and subtropical regions on Earth, which receive nutrient-rich aquaculture effluent from nearby farming activities and accumulated organic contaminnat from industrial wastewater discharge (Gomes Gomes et al., 2008; Giri et al., 2011). The highly productive and diverse microbial community living in tropical and subtropical mangrove ecosystems continuously transforms varied nutrients into sources of nitrogen, phosphorus, and other nutrients that can be used by the plants (Ouyang et al., 2017; Yang et al., 2017). Due the ability to absorb waste and pollutants, mangroves are considered as significant sinks for pollutants from freshwater discharges as well as from contaminated tidal water (Marchand et al., 2016). Therefore, mangrove sediments are suitable for exploring MG degrading microorganisms because of input of carbon in the form of litter which then acts as a substrate for decomposition by microbe. The bioremediation potential of microorganisms isolated from hydrocarbon-contaminated environments was as active as or even higher than those originating from non-contaminated sediments (Jones et al., 2011). For now, a few culturable microorganisms with the degradation activity of dyestuff pollutant are isolated from mangrove sediment microbiome. Two strains, P1 and D1, isolated from mangrove sediments previously, were found with the decolorization ability against a variety of dyes, including nitomill brill crimson, methyl red, and nitro green B (Srinivasan et al., 2014). A mangrove-derived strain, *Aplanochytrium* sp., was reported with MG degrading activity which could remove 86.32% of MG within 5.5 days (Gomathi et al., 2013). Despite global advancement in understanding the microbial diversity in mangrove sediments, more than 90% of environmental microorganisms remain unculturable (Amann et al., 1990; Schloss and Handelsman, 2005; Kimura, 2006). MG degradation potential could be difficult to be assessed and utilized by using traditional culture-dependent method.

Nowadays, culture-independent metagenomic library has successfully used for discovering of novel biosynthetic gene from diverse environments. Lac15, a laccase isolated from marine bacterial metagenome, could degrade several industrial dyes belonged to reactive azo class under alkalescent conditions (Fang et al., 2011). Besides, a novel bacterial laccase Lac21 was isolated from metagenomic library of the South China Sea. Lac21 could remove 80% of Reactive Deep Blue M-2GE (50 mg/L) within 24 h (Fang et al., 2012). However, the reliance on relatively low-throughput of clone libraries combined with activity-based screening could limit screening output (Pope and Moran, 2010; Hess et al., 2011). In addition, metagenomic library focuses on the individual functional gene, not conveniently provides the capacity and diversity of the functional gene in a certain environmental sample. Sequence-based screening of metagenomics combined with the databases, such as Kyoto Encyclopedia of Genes and Genomes (KEGG), Cluster of Orthologous Groups of Proteins (COG), etc., provides a high-performance method on screening the sequences and abundance of potential functional genes (Hugenholtz and Tyson, 2008; Simon and Daniel, 2009). For now, several MDGs have been isolated from MG-degrading strains and metagenomics libraries, including laccase (LACC) (Murugesan et al., 2009), peroxidase (POD) (Ulson de Souza et al., 2007), cytochrome p450 (CYP) (Jefferson and Jones, 2003), triphenylmethane reductase (TMD) (Kim et al., 2008) and triphenylmethane dye oxidase (TpmD) (Ren et al., 2006). However, MDGs resources and abilities from different environment, such as mangrove sediment, were still cryptical. Moreover, the enzymatic properties of MDGs from varied gene families, such as degrading activity, stability against complex conditions, and more radical degradation products, are still strongly desired.

In this study, the culture independent metagenomic method was applied in understanding bacterial abundance and diversity, assessing the gene families, and revealing the novel MDGs genes of the mangrove sediment along South China Sea. Based on the metabolism pathway analysis, some potential MDGs from different gene families were selected and prokaryotic expressed. The biochemical characterization of the corresponding enzymes of MDGs, including temperature, pH, metal ions, metal-chelator, salinity, ionic detergent, and degradation pathway, were tested to further analyze their adaptability and potential to serve as tool enzymes in MG bioremediation.

## Materials and Methods

### Sampling and Environment DNA (eDNA) Extraction

The sediment samples were collected from mangrove forest located in Longhai City, Fujian, China (Zini Mangrove Nature Reserve, 24°20′N, 117°45′E). About 50 g of sediments were collected in a 50 mL of sterile centrifugal tube and stored on ice. Stored the sediments samples in ultra-low temperature freezer (-80°C) after they were brought back to lab.

eDNA was extracted using the chemical lysis and enzyme digestion method. It was purified with low melting-point agarose [Sangon Biotech (Shanghai) Co., Ltd., China] and agarase (Takara Biotechnology Co., Ltd., Japan). Three replications of extraction and purification were performed and pooled together to avoid the extraction biases.

### Sequencing and Bioinformatics Analysis of Metagenome

eDNA sample was used for shotgun paired-end library construction. DNA sample was break into the fragments with the length of about 400 bp using ultrasonic breakers (Covaris, United States). End-repair, adaptor jointing, and purification of the DNA fragments were performed to construct the paired-end library. The concentration of the library was measured by Qubit 2.0 Fluorometer (Invitrogen, United States). High-throughput sequencing using Illumina HiSeq 2500 (Illumina Inc., United States) was performed by Shanghai Majorbio Bio-Pharm Technology Co. (China). Approximately 10 GB of data were generated and the raw reads containing “N” or adaptors were removed from the dataset and the clean reads were retaining for further analysis. The raw data of Illumina sequencing has been deposited in Sequence Read Archive (SRA) database^1^ under the accession numbers SRR5824292.

All clean reads were assembled using SOAPdenovo with Kmer of 43–47. The best Kmer was identified on the basis of contig numbers, contig N50, contig length, etc. MetaGeneMark was used to predict the open reading frames (ORFs) based on the contigs and singletons obtained. ORFs were aligned using BLAST+ in Nr database of NCBI (cut-off E-value of 1e-5) and were annotated with the functional information. The putative MDGs mentioned before were filtered out according to the gene annotation. The bacteria origin of putative MDGs was predicted based on sequence homology with the protein sequences in NCBI Nr database using BLAST+ (cut-off E-value of 1e-5). COGs functional classification was conducted in STRING database^2^ and KEGG^3^ was used to obtain the biological pathways with a BLAST algorithm in KEGG database. All reads were aligned in SSU rRNA database of SILVA (cut-off E-value of 1e-5) to investigate taxonomic assignment of sampling site.

### Full-Length Amplification of *mgv-laccase* and *mgv-p450* Genes

Due to relatively lower similarities (about 50% similarities) with known sequences compared with other putative MDGs, three representative MDGs belonging to POD, LACC, and CYP, respectively, were chosen to be expressed in *E. coli* BL21 (DE3) cells and their MG-degrading activities were further detected. Three genes were labeled as *mgv-peroxidase*, *mgv-laccase*, and *mgv-p450*, respectively. Among them, *mgv-laccase* and *mgv-p450*, which had not complete ORFs, were amplified by TAIL-PCR with Genome Walking Kit (Takara Biotechnology Co., Ltd., Japan) according to the instructions. In brief, eDNA solution was used as the template for TAIL-PCR. Thermal asymmetric cycle was performed for three times, and the programs and reagent doses were mixed strictly according to the instructions. The degenerate primers for thermal asymmetric amplification were provided in the kit, and the specific primers (**Table 1**) for *mgv-laccase* and *mgv-p450* were synthesized by Invitrogen (Shanghai) Co., Ltd. (United States). The possible DNA fragments were retrieved with TIANgel Midi Purification Kit (Tiangen Biotech Co., Ltd., China) after 1% agarose gel electrophoresis, and were cloned into pMD19-T Vector (Takara Biotechnology Co., Ltd., Japan). Then, DNA sequencing was conducted by using 3730xl DNA Analyzer (Thermo Fisher Scientific Co., Ltd., United States). Sequences were assembled with DNAman (Version 6.0.3.99) and ORFs of *mgv-laccase* and *mgv-p450* were predicted in ORF Finder^4^.

**Table 1:** Primers for TAIL-PCR and expression of malachite green-degrading genes (MDGs).

Primer name	Sequence (5′–3′)	Restriction enzyme
Laccase SP1	TACGCAATGGTGGCAGCAGTCGG	–
Laccase SP2	CCGCAGTCGCCAGGGCTTTTGAT	–
Laccase SP3	CGGTCTTCTCGGGCGAACAGTCA	–
P450-F-SP1	ATTTTCCCGGCTTCCCGATGACT	–
P450-F-SP2	CCTATTTCGTGAACGATCCCGACG	–
P450-F-SP3	ACGTTCGCGGACGATGATCCAG	–
P450-R-SP1	AGCATCTTGACGCGCTCGAACC	–
P450-R-SP2	ACCATGCTGACAAGGTCCCCGTAC	–
P450-R-SP3	GTCATCGGGAAGCCGGGAAAAT	–
Mgv-rPOD -F	ACGGCGAATTCTCCTGGATTGACGAGATTGAAATCGACGAA	*Eco*RI
Mgv-rPOD -R	ATTCTGGTCGACGGTCGTTGAGATAGCCCGCGACTTCCT	*Sal*I
Mgv-rLACC-F	TACTGAATTCAAAACAGTTAAACAAGGTAAAATCCATTACCT	*Eco*RI
Mgv-rLACC-R	CATGCTCGAGTACGCAGTAAAGCAAACCCCATCTGG	*Xho*I
Mgv-rP450-F	GATAGAATTCACTCAACCCCAGCGCGCAATC	*Eco*RI
Mgv-rP450-R	CATAGTCGACGTTTTCCCGGCTTCCCGATGACTTG	*Sal*I

*The bases underlined were restriction enzyme cutting site*.

### Expression and Purification of MDGs

Three putative MDGs, *mgv-laccase*, *mgv-p450*, and *mgv-peroxidase*, were cloned by primers (**Table 1**) designed according to ORFs. The PCR product was ligated into pET-32a vector (Novagen Inc., United States) and transformed into *E. coli* BL21 (DE3) cells. Recombination proteins were purified through nickel-affinity chromatography column. The three recombinant proteins were named Mgv-rPOD, Mgv-rLACC, and Mgv-rCYP, respectively. Sequences of *mgv-laccase*, *mgv-p450*, and *mgv-peroxidase* have been deposited in GenBank database under the accession numbers MF461728, MF461729, and MF461730, respectively.

### Biochemical Analysis of MG-Degrading Activity of Recombinant Proteins

The MG-degrading activity of recombinant proteins was measured by the decolorization efficiency of MG with the formulae as follows: Decolorization efficiency (%) = (A - B)/A × 100, where A is the initial absorbance of MG and B is the final absorbance of MG at a wavelength of 622 nm. To value the cost of MG degradation, same quality (100 μg) of the three proteins were used in decolorization experiment.

The effect of temperature on the decolorizing efficiency was determined by measuring the decolorization percentage of MG at 10°C, 20°C, 30°C, 40°C, 50°C, 60°C, and 70°C, respectively. Similarly, the effect of pH was determined in the buffer solution of citric acid/sodium citrate (pH 3.0–6.0), phosphate (pH 6.0–8.0), Tris-HCl (pH 8.0–10.0). The effect of metal ions was determined with ZnSO_4_, MgSO_4_, MnSO_4_, NiSO_4_, FeSO_4_, CuSO_4_, Al_2_(SO_4_)_3_, and CaSO_4_ (1 mM). The effect of the potential inhibitors was determined with NaCl (1, 10, and 100 mM), EDTA (1, 10, and 100 mM) and SDS (0.1, 0.5, and 1%).

### Determination of MG Biodegradation Intermediates

The method for the determination of MG biodegradation intermediates referred to the previous studies (Du et al., 2011, 2013; Wang et al., 2012; Yang et al., 2015). To determine the MG biodegradation intermediates, 20 mg/L MG was mixed with the three recombination proteins and incubated at 30°C for 12 h. The products were desalted with C18 solid phase extraction column (Waters Sep-pak, United States). All the samples were analyzed using LC-MS (UPLC-Tof MS System, Waters, United States). The mobile phase contained H_2_O and acetonitrile. The initial proportion of acetonitrile was 5% and reached 100% within 15 min. The injection volume was 10 μl, and other parameters were set at the default settings.

## Results

### Metagenomic Analysis

**FIGURE 1:**
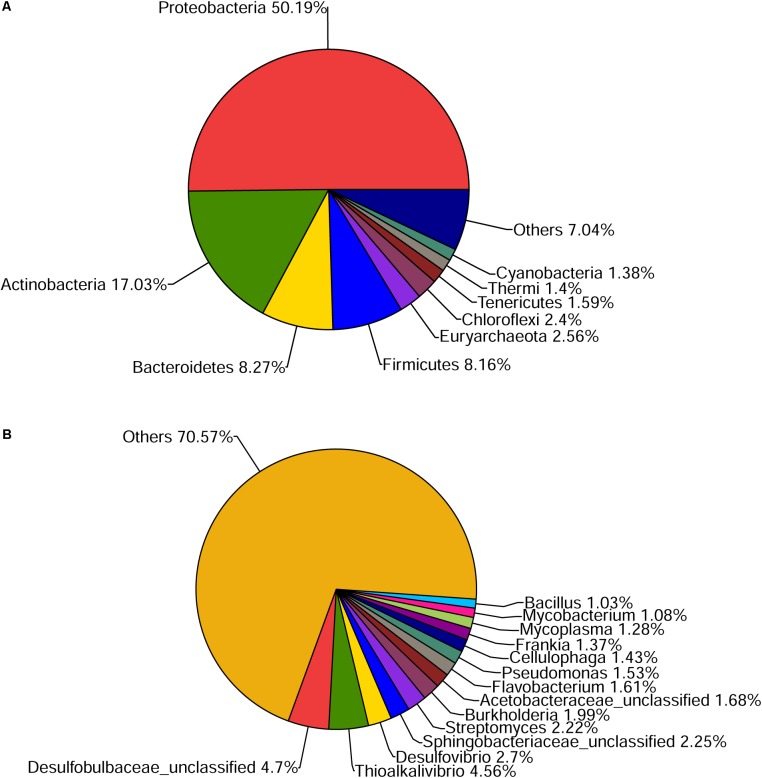
Community composition of mangrove sediment microbiome on phylum **(A)** and genus level **(B)** revealed by the SSU rRNA genes in the dataset.

Approximately 10 GB of data was generated and 9.4 GB was remained as clean data. Subsequently, 33,756 contigs were assembled with best Kmer of 45 (**Supplementary Table S1**) and 44,743 ORFs were predicted. Based on SSU rRNA genes from metagenomic sequencing, microbial community showed great diversity on phylum level, mainly including *Proteobacteria* (50.19%) and *Actinobacteria* (17.03%), followed by other minor groups (**Figure 1A**). On genus level, a total of 666 bacterial genera were found in our datasets. Due to the lack of adequate contig length, 70.57% genus of total microbes remained unknown (**Figure 1B**). Twenty-three COG categories (**Supplementary Figure S3**) and 258 pathways in KEGG (**Supplementary Table S2**) were found. Thirteen metabolic pathways of common pollutant, such as styrene, ethylbenzene, aromatic compounds, chlorinated cyclohexane, and benzene degradation, were also detected in KEGG analysis.

### Scanning, Cloning, and Expression of Putative MDGs

**FIGURE 2:**
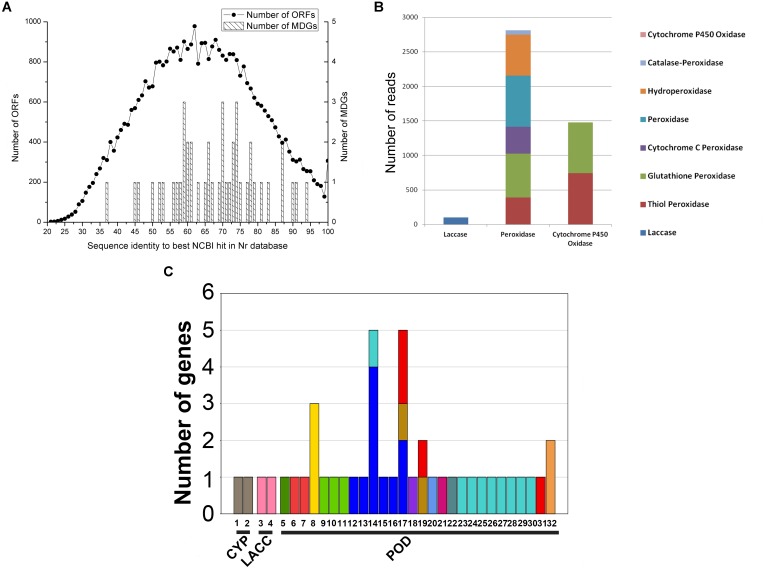
Bioinformatics analysis of putative malachite green-degrading genes (MDGs). **(A)** Distribution of best identity in Nr database of all open reading frames (ORFs) and putative MDGs. **(B)** The number of 44 putative MDGs in the dataset of mangrove sediment microbiome. **(C)** The bacteria origin of putative MDGs. The numbers on the *X*-axis indicated the genera of *Nitrososphaera, Acidovorax, Desulfuromonas, Chromobacterium, Geopsychrobacter, Cellvibrio, Shewanella, Simiduia, Oscillatoria, Thiothrix, Thiocapsa, Draconibacterium, Frankia, Nitrosomonas, Sedimenticola, Bizionia*, NO-TaxID*, Formosa*, norank, *Leucothrix, Magnetococcus, Spirochaeta, Rhodanobacter, Gemmobacter, Kangiella, Gaetbulibacter, Mariniradius, Zooshikilla, Imtechella, Salinibacter, Gelidibacter*, and *Marinobacter*, respectively.

Forty-four putative MDGs were found in 33,756 contigs with 55–75% identities in Nr database and belonged to three gene families (**Supplementary Table S4** and **Figure 2A**). *Peroxidase* genes possessed dominant proportion (up to 93.54% of 44 putative MDGs), followed by LACC (3.40%) and CYP (3.06%) (**Figure 2B**). Of them, seven genes were unclassified because of their unavailable TaxIDs and the remaining 37 genes were assigned to 30 bacterial genera (**Figure 2C**). Combined with the analysis of microbial communities, the corresponding functional genes related to the pollutant degradation and their possible bacterial origin were listed in **Supplementary Table S5**. Amongst these microorganisms, most (18/30) genera belonged to *Proteobacteria*, followed by *Bacteroidetes* (8/30), *Actinobacteria* (1/30), *Spirochaetes* (1/30), *Thaumarchaeota* (1/30), and *Cyanobacteria* (1/30) (**Supplementary Table S5**).

Phylogenetic tree based on the amino acid sequences showed that *mgv-laccase* clustered within LACC of *Pelobacter seleniigenes* (**Supplementary Figure S1A**), *mgv-p450* gathered with CYP of *Salinisphaera shabanensis* (**Supplementary Figure S1B**) and *mgv-peroxidase* was close to POD from *Salinibacter ruber* (**Supplementary Figure S1C**).

**FIGURE 3:**
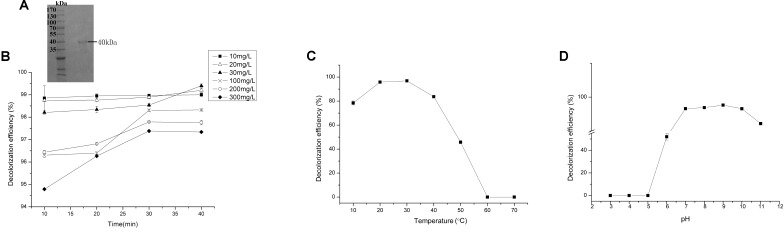
Malachite green (MG) degradation characteristics of Mgv-rPOD. **(A)** SDS-PAGE result of Mgv-rPOD. **(B)** The effect of MG concentration on the MG decolorization efficiency of Mgv-rPOD. **(C)** The effect of temperature on the MG decolorization efficiency of Mgv-rPOD. **(D)** The effect of pH on the MG decolorization efficiency of Mgv-rPOD.

**FIGURE 4:**
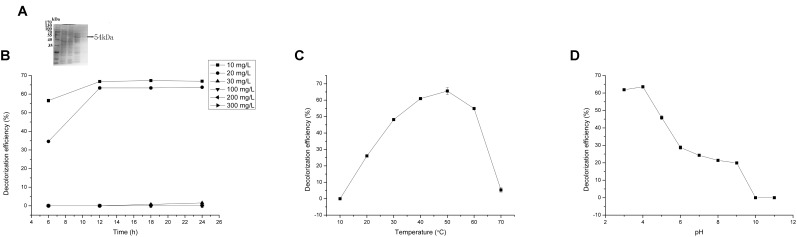
Malachite green (MG) degradation characteristics of Mgv-rLACC. **(A)** SDS-PAGE result of Mgv-rLACC. **(B)** The effect of MG concentration on the MG decolorization efficiency of Mgv-rLACC. **(C)** The effect of temperature on the MG decolorization efficiency of Mgv-rLACC. **(D)** The effect of pH on the MG decolorization efficiency of Mgv-rLACC.

**FIGURE 5:**
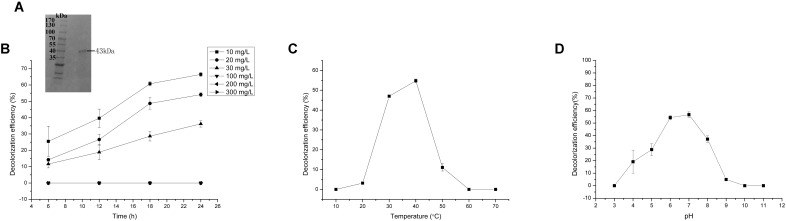
Malachite green (MG) degradation characteristics of Mgv-rCYP. **(A)** SDS-PAGE result of Mgv-rCYP. **(B)** The effect of MG concentration on the MG decolorization efficiency of Mgv-rCYP. **(C)** The effect of temperature on the MG decolorization efficiency of Mgv-rCYP. **(D)** The effect of pH on the MG decolorization efficiency of Mgv-rCYP.

Reasonable ORFs were found in both *mgv-laccase* and *mgv-p450* by TAIL-PCR and sequencing. The length of complete *mgv-laccase* gene and *mgv-p450* gene was 795 bp and 627 bp, respectively. Mgv-rPOD, Mgv-rLACC and Mgv-rCYP were purified with molecular weight of 40 kDa (**Figure 3A**), 54 kDa (**Figure 4A**), and 43 kDa (**Figure 5A**), respectively.

### MG-Degrading Characteristics Analysis

**FIGURE 6:**
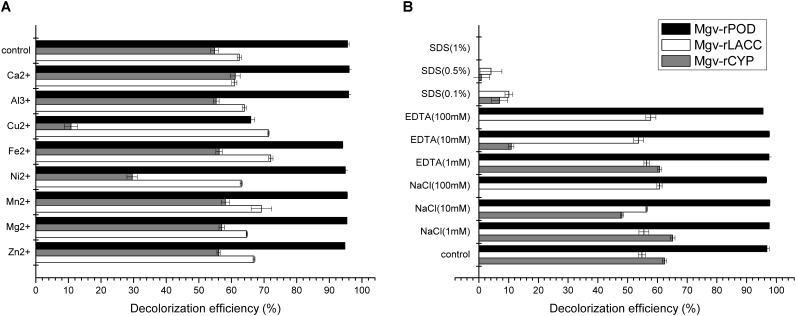
The effect of metal ions **(A)** and potential inhibitors **(B)** on the MG decolorization efficiency of Mgv-rPOD, Mgv-rLACC, and Mgv-rCYP.

Mgv-rPOD showed strong MG-degrading activity. After incubated with Mgv-rPOD for only 40 min, the MG decolorization efficiency could reach 99.0%, 99.2%, 99.4%, 96.3%, 97.8%, and 97.3% in 10 mg/L, 20 mg/L, 30 mg/L, 100 mg/L, 200 mg/L, and 300 mg/L of MG, respectively (**Figure 3B**). The Mgv-rPOD had the optimum temperature 30°C for color removal with 96.9% of MG-decolorizing efficiency and still maintained the high MG-degrading activity at 20°C (**Figure 3C**). Also, experimental results showed that high MG decolorizing activity was often between 7.0 and 10.0 and the observed optimum pH for the reaction was 9.0 (**Figure 3D**). Cu^2+^ had obviously negative effect, which caused the loss of 65.9% MG-degrading activity of Mgv-rPOD. Meanwhile, lots of bubbles were generated in the reaction with Cu^2+^. Besides, the use of other metal ions did not show much variation in the activity of decolorizing MG (**Figure 6A**). Different concentrations of EDTA (1, 10, and 100 mM) and NaCl (1, 10, and 100 mM) could not affect the decolorizing efficiency. However, there was a sharp decrease with 0.1% SDS and an absence of MG-degrading activity with 1% SDS (**Figure 6B**).

Mgv-rLACC could decolorize 66.9% and 63.7% of MG at a concentration of 10 mg/L and 20 mg/L (**Figure 4B**) in 24 h. However, in higher concentration of MG, Mgv-rLACC did not show obvious MG-decolorizing characteristic. The optimum pH of Mgv-rLACC was 4.0 (**Figure 4D**) and the optimum temperature was 50°C (**Figure 4C**). 10 mM and 100 mM sodium chloride additive dramatically inhibited the decolorizing activity of MG, up to 48.1% and 0%, respectively (**Figure 6B**). Metal-chelators, EDTA, had a negative impact on Mgv-rLACC (**Figure 6B**) due to the lack of Cu^2+^ which may serve as an electron transfer during MG degradation. There were no significant differences observed in the variation of metal ions (**Figure 6A**).

With increasing of the MG concentration, the Mgv-rCYP showed the trend of gradual reduction. The rates of MG degradation incubated with MG concentration of 10 mg/L, 20 mg/L, and 30 mg/L for 24 h were 66.5%, 54.1%, and 36.2%, respectively (**Figure 5B**). As Mgv-rLACC, no decolorizing phenomenon was detected with higher concentration of MG (**Figure 5B**). The optimum pH of Mgv-rCYP for MG degradation was 7.0 (**Figure 5D**) and the optimum temperature was 40°C (**Figure 5C**). Cu^2+^, Ni^2+^, and SDS could inhibit MG-degrading activities significantly (**Figures 6A,B**). MG-degrading characteristics of three recombination proteins were comparatively analyzed (**Supplementary Table S3**).

### Intermediates of MG Degradation by the Three Recombination Proteins

**FIGURE 7:**
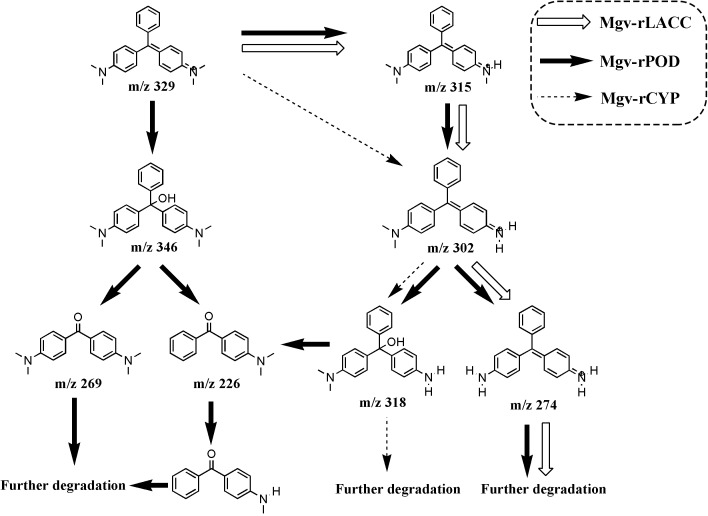
The putative MG degradation pathway deduced from the products of Mgv-rPOD, Mgv-rLACC, and Mgv-rCYP. The intermediates connected by the black, white and dashed arrows indicated the MG-degrading pathway of Mgv-rPOD, Mgv-rLACC, and Mgv-rCYP, respectively.

By using LC-MS, different intermediates were detected in the MG-degrading process of three enzymes, respectively. Three intermediates from Mgv-rLACC according to the m/z values, were desmethyl-MG (m/z 315), didesmethyl-MG (m/z 302) and tetradesmethyl-LMG (m/z 274). Mgv-rCYP degraded MG into two products, didesmethyl-MG (m/z 302) and hydroxyl(didesmethyl)-MG (m/z 318). The intermediate products from Mgv-rPOD were the most diverse, including Desmethyl-MG (m/z 315), didesmethyl-MG (m/z 302), hydroxyl(didesmethyl)-MG (m/z 318), tetradesmethyl-LMG (m/z 274), hydroxyl-MG (m/z 346), Michler’s ketone (m/z 269), 4-(dimethylamino) benzophenone (m/z 226), and 4-(methylamino) benzophenone (m/z 212) (**Figure 7** and **Supplementary Figure S2**).

## Discussion

Advanced metagenomic sequencing has been widely used in studying the structures and functions of microbial communities (Andreote et al., 2012; Soares et al., 2017). There were evidences from metagenomics analysis showed that abundant microorganisms and genes existing in mangrove environment could be implicated in the process of pollutant degrading (Andreote et al., 2012; Gonçalves et al., 2015). In this study, a broad diversity of bacterial and MDGs profiles were confirmed in mangrove sediments. However, the actual bioactivities of the functional genes were still unclear due to the lack of characteristics of complete gene products. Metagenomics based TAIL-PCR was an effective and relatively low-cost method for obtaining complete genes. The results that MDGs from different gene families were expressed and the corresponding MG-degrading characteristics were tested to further prove the validity of the strategy.

Mangroves are one among the world’s most productive ecosystems and are of ecological, economic, and societal importance (Mumby et al., 2004; Donato et al., 2011). Microbes play a key role in maintaining this productivity and conserve this ecosystem (Andreote et al., 2012). From our dataset of mangrove sediments, 666 bacterial genera (**Figure 1**) and 13 pollutant degradation pathways (**Supplementary Table S2**) were found. Genes that were found more frequently in one community than others are assumed to endow beneficial function on that community (Hugenholtz and Tyson, 2008). It was implied that microbes in the mangrove could constitute a large gene pool related to pollutant transformation with potential biotechnological and environmental implications. Besides, the apparent associations between various bacterial taxa and functional genes were analyzed (**Figure 2D**). Thirteen genera were identified to be the original genera of the 44 MDGs. Of them, 12 genera have been reported to be involved in organic pollutant degradation. *Nitrosomonas* could be the most active genera with the most diverse MDGs, including four *glutathione peroxidase* genes and one *peroxidase* gene. It was reported that the genus had significant activities to degrade halogenated aliphatic (Wahman et al., 2005), trichloroethylene (Suttinun et al., 2010) and PAHs (Chang et al., 2002). The *Shewanella* genus could also remove MG, methyl violet B (Chen et al., 2010), naphthylamine sulfonic azo dye (Hong et al., 2007) and naphthol green B dye (Xiao et al., 2012). This information provided important clues to discover microbial and genetic resources from the mangroves. According to the previous studies, lots of members of *Proteobacteria* and *Bacteroidetes* harbor many functional genes, including biodegradation genes, phenol degradation genes and PAHs degradation genes (Klankeo et al., 2009; Fang et al., 2013; Zhao et al., 2016). However, the functions of *Proteobacteria* and *Bacteroidetes* for MG degradation have been poorly studied. In our work, 18/30 and 8/30 of those bacterial origin, respectively, belonged to the phyla of *Proteobacteria* and *Bacteroidetes*, which indicated that *Proteobacteria* and *Bacteroidetes* also played a crucial role of MG degradation in mangrove sediments.

Forty-four genes were predicted to be related to MG-degrading and belonged to three gene classes (LACC, POD, and CYP, **Figure 2C**). Relative lower amino acid identities from 50% to 70% (**Figure 2B**) suggested most genes were new sequences which were not reported in other environments. The diversity and richness of functional gene represented by the metagenomes implied the potential of mangrove microorganisms in environment restoration. Three MDGs from three gene families were expressed and the MG-degrading characteristics from the corresponding recombinant proteins at varied testing levels were verified. Mgv-rPOD showed the highest MG-degrading activity among the three MG-degrading enzymes (**Figure 3**). Due to the usual low temperature of natural seawater (about 10–35°C), the low-temperature adaptability of Mgv-rPOD which kept a high degradation activity (95.8%) at 20°C, has great advantage applying in marine environment. A strong anti-interference ability to environmental factors, including the adaptabilities to metal ions (except Cu^2+^), metal-chelator (represented by EDTA), and the stability in different salinity further showed that Mgv-rPOD could be a promising bio-resource for removing MG from complex wastewaters. In addition, Mgv-rPOD had advantages in MG-degrading activity over not only the enzymes from this study but also other peroxidases reported in previous works. A manganese peroxidase, which showed the MG-degrading activity, was discovered from the white rot fungus *Irpex lacteus* F17. The manganese peroxidase could efficiently degrade 96% of MG at the concentration of 200 mg/L within 1 h; however, only about 75% of MG was degraded by using this enzyme at the MG concentration of 300 mg/L (Yang et al., 2016). By comparison, Mgv-rPOD could degrade more than 97% of MG within 30 min at the concentration of 300 mg/L, and the concentration was higher than most MG-degrading enzymes (Li et al., 2009; Saravanakumar et al., 2013; Zhang et al., 2013). Besides, many MG-degrading enzymes could only work at a narrow pH range. For instance, Saravanakumar et al. (2013) isolated manganese peroxidase isozyme H4 from *Phanerochaete chrysosporium.* However, about 50% MG-degrading activity of the enzymes was lost when the working pH was deviated from the optimum pH 4.5 (Saravanakumar et al., 2013). Unfortunately, it is very difficult to ensure the pH of nature environment when this enzyme was used in the remediation of MG pollution. Conversely, Mgv-rPOD possessed a wider pH range (7.0–11.0) for MG degradation, which suggested that Mgv-rPOD was more suitable for practice abatement of MG pollution. The possible mechanisms of MG degradation by Mgv-rPOD, Mgv-rLACC, and Mgv-rCYP were speculated according to the intermediates detected by LC-MS (**Figure 7**). All the processes of MG-degrading by the three recombination proteins were begin with a series of *N*-demethylation reactions, which was consistent with the former reports (Kedderis and Hollenberg, 1983; Cha et al., 2001; Murugesan et al., 2009). The products degrading by Mgv-rLACC and Mgv-rCYP were retained the triphenyl structure, and no further intermediates were detected, which was consistent with the previous study (Cha et al., 2001; Murugesan et al., 2009). The products of Mgv-rPOD suggested a further process of MG degradation. Mgv-rPOD not only mediated the *N*-demethylation but could also disconnect the triphenyl structure. Therefore, Mgv-rPOD was an attractive option for the remediation of MG pollution due to the high MG-degrading activity, high anti-interference, wide pH working condition, and the more complete degradation process.

## Conclusion

Three recombinant proteins, especially Mgv-rPOD, were discovered and characterized as promising enzymes for the remediation of MG pollution in nature environment. Meanwhile, metagenomics provided an efficient path to view the microbial community structure and the functional (metabolic) potential of microbial community. The study further confirmed not-yet-cultivated bacteria from mangrove environments were a potential source for novel biocatalysts.

## Author Contributions

WQ and JZ designed this work and wrote this paper. WQ, TL, and JZ performed the experiments in this work. DW and TL collected the sediment samples. GH revised this paper.

## Conflict of Interest Statement

The authors declare that the research was conducted in the absence of any commercial or financial relationships that could be construed as a potential conflict of interest.

## Footnotes

1 https://www.ncbi.nlm.nih.gov/sra

2 http://string-db.org/

3 http://www.genome.jp/kegg/

4 http://www.ncbi.nlm.nih.gov/projects/gorf/
