# Supplementary material for: Metagenomics-Based Discovery of Malachite Green-Degradation Gene Families and Enzymes From Mangrove Sediment
Source: Front Microbiol. 2018 Sep 11;9:2187. doi: 10.3389/fmicb.2018.02187 (PMC6143792; doi:10.3389/fmicb.2018.02187)
Supplement: Supplementary file 1 [file Data_Sheet_1.PDF]

Supplementary Material:

Metagenomics-based discovery of malachite green-degradation gene families and enzymes from mangrove sediment

Wu Qu [2], Tan Liu[1], Dexiang Wang [1], Guolin Hong [3], Jing Zhao [1]*

[1] *College of Ocean and Earth Science of Xiamen University，Xiamen, 361005, China*

[2] *School of Life Sciences, Xiamen University, Xiamen, 361005, China*

[3]*The department of laboratory medicine, the First Affiliated Hospital of Xiamen University, Xiamen, 361005, China*

*Corresponding author: Jing Zhao

Address: Xiangan District, Zhoulongquan Building, Xiamen University

Tel.: +86-592-2880811；Fax: +86-592-2880172;

E-mail address: sunnyzhaoj@xmu.edu.cn

ORCID: 0000-0003-1895-7620

Wu Qu

Tel.: +86-592-2880172; Fax: +86-592-2880172;

E-mail address: quwu2339525@126.com

Tan Liu

Tel.: +86-592-2880172; Fax: +86-592-2880172;

E-mail address: 394536091@qq.com

Dexiang Wang

Tel.: +86-592-2880172; Fax: +86-592-2880172;

E-mail address: dxwang@xmu.edu.cn

Guolin Hong

Tel.: +86-592-2139507; Fax: +86-592-2880172;

E-mail address: 18860089899@139.com

1 Supplementary Figures and Tables

1.1 Supplementary Tables

Table S1 Statistics of assembly result with each Kmer

Sample	Kmer	Contig_num	Contig_length_all	Contig_N50	Contig_N90	Contig_max	Contig_min
Mgv	K41	41801	27538174	696	369	12162	300
Mgv	K43	37850	25159839	706	372	12310	300
Mgv	K45	33756	22670283	718	376	12162	300

Table S2 KEGG pathways statistics

Pathway	Pathway Definition	Number of Sequences
path:ko00830	Retinol metabolism	36
path:ko00650	Butanoate metabolism	304
path:ko04911	Insulin secretion	1
path:ko05030	Cocaine addiction	6
path:ko00945	Stilbenoid, diarylheptanoid and gingerol biosynthesis	1
path:ko03430	Mismatch repair	288
path:ko00760	Nicotinate and nicotinamide metabolism	128
path:ko04261	Adrenergic signaling in cardiomyocytes	1
path:ko05203	Viral carcinogenesis	23
path:ko04974	Protein digestion and absorption	14
path:ko03060	Protein export	228
path:ko01054	Nonribosomal peptide structures	6
path:ko00072	Synthesis and degradation of ketone bodies	48
path:ko03440	Homologous recombination	312
path:ko04112	Cell cycle – Caulobacter	225
path:ko00930	Caprolactam degradation	42
path:ko00473	D-Alanine metabolism	47
path:ko00430	Taurine and hypotaurine metabolism	81
path:ko01200	Carbon metabolism	1595
path:ko04932	Non-alcoholic fatty liver disease (NAFLD)	23
path:ko00513	Various types of N-glycan biosynthesis	1
path:ko00130	Ubiquinone and other terpenoid-quinone biosynthesis	130
path:ko03410	Base excision repair	150
path:ko05160	Hepatitis C	1
path:ko05020	Prion diseases	4
path:ko00240	Pyrimidine metabolism	769
path:ko00900	Terpenoid backbone biosynthesis	155
path:ko00624	Polycyclic aromatic hydrocarbon degradation	19
path:ko00051	Fructose and mannose metabolism	130
path:ko04621	NOD-like receptor signaling pathway	25
path:ko05152	Tuberculosis	67
path:ko05012	Parkinson's disease	22
path:ko04011	MAPK signaling pathway – yeast	9
path:ko04120	Ubiquitin mediated proteolysis	1
path:ko00472	D-Arginine and D-ornithine metabolism	3
path:ko00984	Steroid degradation	8
path:ko04920	Adipocytokine signaling pathway	54
path:ko00520	Amino sugar and nucleotide sugar metabolism	206
path:ko05215	Prostate cancer	25
path:ko00040	Pentose and glucuronate interconversions	67
path:ko00450	Selenocompound metabolism	176
path:ko00620	Pyruvate metabolism	487

Table S2 (continued) KEGG pathways statistics

Pathway	Pathway Definition	Number of Sequences
path:ko02060	Phosphotransferase system (PTS)	22
path:ko00310	Lysine degradation	198
path:ko04973	Carbohydrate digestion and absorption	10
path:ko05031	Amphetamine addiction	6
path:ko00362	Benzoate degradation	96
path:ko00010	Glycolysis / Gluconeogenesis	375
path:ko04612	Antigen processing and presentation	25
path:ko00640	Propanoate metabolism	391
path:ko03013	RNA transport	37
path:ko03070	Bacterial secretion system	301
path:ko03030	DNA replication	255
path:ko00633	Nitrotoluene degradation	82
path:ko05169	Epstein-Barr virus infection	4
path:ko04940	Type I diabetes mellitus	34
path:ko01120	Microbial metabolism in diverse environments	2290
path:ko04918	Thyroid hormone synthesis	19
path:ko00591	Linoleic acid metabolism	17
path:ko04210	Apoptosis	3
path:ko00790	Folate biosynthesis	151
path:ko01110	Biosynthesis of secondary metabolites	2978
path:ko04070	Phosphatidylinositol signaling system	28
path:ko04728	Dopaminergic synapse	6
path:ko00052	Galactose metabolism	86
path:ko00660	C5-Branched dibasic acid metabolism	89
path:ko00523	Polyketide sugar unit biosynthesis	20
path:ko04113	Meiosis – yeast	37
path:ko00970	Aminoacyl-tRNA biosynthesis	522
path:ko00643	Styrene degradation	41
path:ko00983	Drug metabolism - other enzymes	101
path:ko04111	Cell cycle – yeast	1
path:ko00380	Tryptophan metabolism	220
path:ko00260	Glycine, serine and threonine metabolism	434
path:ko00300	Lysine biosynthesis	159
path:ko00053	Ascorbate and aldarate metabolism	36
path:ko03020	RNA polymerase	139
path:ko00531	Glycosaminoglycan degradation	4
path:ko01100	Metabolic pathways	710
path:ko01210	2-Oxocarboxylic acid metabolism	339
path:ko05132	Salmonella infection	10
path:ko01055	Biosynthesis of vancomycin group antibiotics	7
path:ko05150	Staphylococcus aureus infection	2
path:ko04976	Bile secretion	6

Table S2 (continued) KEGG pathways statistics

Pathway	Pathway Definition	Number of Sequences
path:ko04914	Progesterone-mediated oocyte maturation	25
path:ko00562	Inositol phosphate metabolism	46
path:ko00071	Fatty acid degradation	269
path:ko00903	Limonene and pinene degradation	88
path:ko00710	Carbon fixation in photosynthetic organisms	223
path:ko00600	Sphingolipid metabolism	52
path:ko02020	Two-component system	697
path:ko04919	Thyroid hormone signaling pathway	1
path:ko05168	Herpes simplex infection	2
path:ko00670	One carbon pool by folate	200
path:ko04970	Salivary secretion	1
path:ko05110	Vibrio cholerae infection	1
path:ko02030	Bacterial chemotaxis	57
path:ko04910	Insulin signaling pathway	50
path:ko01051	Biosynthesis of ansamycins	20
path:ko00590	Arachidonic acid metabolism	33
path:ko00603	Glycosphingolipid biosynthesis - globo series	2
path:ko04110	Cell cycle	1
path:ko00623	Toluene degradation	65
path:ko04068	FoxO signaling pathway	27
path:ko03022	Basal transcription factors	4
path:ko00965	Betalain biosynthesis	2
path:ko04142	Lysosome	23
path:ko00550	Peptidoglycan biosynthesis	254
path:ko00950	Isoquinoline alkaloid biosynthesis	33
path:ko03010	Ribosome	723
path:ko00510	N-Glycan biosynthesis	10
path:ko00791	Atrazine degradation	6
path:ko00750	Vitamin B6 metabolism	60
path:ko05034	Alcoholism	6
path:ko00626	Naphthalene degradation	27
path:ko00540	Lipopolysaccharide biosynthesis	162
path:ko05142	Chagas disease (American trypanosomiasis)	20
path:ko04930	Type II diabetes mellitus	19
path:ko00340	Histidine metabolism	196
path:ko00860	Porphyrin and chlorophyll metabolism	228
path:ko03018	RNA degradation	258
path:ko05206	MicroRNAs in cancer	4
path:ko04964	Proximal tubule bicarbonate reclamation	21
path:ko01212	Fatty acid metabolism	380
path:ko03008	Ribosome biogenesis in eukaryotes	18
path:ko00680	Methane metabolism	487

Table S2 (continued) KEGG pathways statistics

Pathway	Pathway Definition	Number of Sequences
path:ko00642	Ethylbenzene degradation	26
path:ko00561	Glycerolipid metabolism	104
path:ko00982	Drug metabolism - cytochrome P450	37
path:ko04726	Serotonergic synapse	6
path:ko00410	beta-Alanine metabolism	165
path:ko00250	Alanine, aspartate and glutamate metabolism	478
path:ko01040	Biosynthesis of unsaturated fatty acids	78
path:ko00312	beta-Lactam resistance	147
path:ko02040	Flagellar assembly	55
path:ko05014	Amyotrophic lateral sclerosis (ALS)	4
path:ko00440	Phosphonate and phosphinate metabolism	10
path:ko03450	Non-homologous end-joining	1
path:ko04626	Plant-pathogen interaction	74
path:ko00622	Xylene degradation	17
path:ko04972	Pancreatic secretion	1
path:ko00780	Biotin metabolism	129
path:ko05410	Hypertrophic cardiomyopathy (HCM)	4
path:ko05010	Alzheimer's disease	42
path:ko05162	Measles	1
path:ko04723	Retrograde endocannabinoid signaling	3
path:ko04917	Prolactin signaling pathway	3
path:ko00253	Tetracycline biosynthesis	49
path:ko00471	D-Glutamine and D-glutamate metabolism	53
path:ko04260	Cardiac muscle contraction	23
path:ko00740	Riboflavin metabolism	64
path:ko00401	Novobiocin biosynthesis	49
path:ko00311	Penicillin and cephalosporin biosynthesis	32
path:ko00908	Zeatin biosynthesis	14
path:ko05120	Epithelial cell signaling in Helicobacter pylori infection	22
path:ko00604	Glycosphingolipid biosynthesis - ganglio series	2
path:ko00351	DDT degradation	5
path:ko00730	Thiamine metabolism	55
path:ko00940	Phenylpropanoid biosynthesis	41
path:ko04915	Estrogen signaling pathway	25
path:ko00627	Aminobenzoate degradation	84
path:ko00364	Fluorobenzoate degradation	12
path:ko00910	Nitrogen metabolism	269
path:ko00121	Secondary bile acid biosynthesis	1
path:ko00195	Photosynthesis	145
path:ko00960	Tropane, piperidine and pyridine alkaloid biosynthesis	46
path:ko05133	Pertussis	13
path:ko05033	Nicotine addiction	1

Table S2 (continued) KEGG pathways statistics

Pathway	Pathway Definition	Number of Sequences
path:ko00511	Other glycan degradation	35
path:ko04614	Renin-angiotensin system	4
path:ko04141	Protein processing in endoplasmic reticulum	57
path:ko00524	Butirosin and neomycin biosynthesis	9
path:ko00906	Carotenoid biosynthesis	6
path:ko05340	Primary immunodeficiency	6
path:ko00480	Glutathione metabolism	184
path:ko00030	Pentose phosphate pathway	202
path:ko00361	Chlorocyclohexane and chlorobenzene degradation	31
path:ko05164	Influenza A	1
path:ko05166	HTLV-I infection	2
path:ko05100	Bacterial invasion of epithelial cells	3
path:ko05416	Viral myocarditis	1
path:ko05146	Amoebiasis	2
path:ko04960	Aldosterone-regulated sodium reabsorption	1
path:ko00281	Geraniol degradation	80
path:ko05205	Proteoglycans in cancer	14
path:ko00140	Steroid hormone biosynthesis	30
path:ko00943	Isoflavonoid biosynthesis	2
path:ko00770	Pantothenate and CoA biosynthesis	168
path:ko05204	Chemical carcinogenesis	24
path:ko00565	Ether lipid metabolism	1
path:ko04727	GABAergic synapse	55
path:ko04724	Glutamatergic synapse	50
path:ko05200	Pathways in cancer	38
path:ko00785	Lipoic acid metabolism	25
path:ko00400	Phenylalanine, tyrosine and tryptophan biosynthesis	236
path:ko00460	Cyanoamino acid metabolism	96
path:ko04146	Peroxisome	114
path:ko00280	Valine, leucine and isoleucine degradation	361
path:ko00625	Chloroalkane and chloroalkene degradation	85
path:ko00290	Valine, leucine and isoleucine biosynthesis	182
path:ko03420	Nucleotide excision repair	197
path:ko00564	Glycerophospholipid metabolism	154
path:ko05111	Vibrio cholerae pathogenic cycle	42
path:ko00500	Starch and sucrose metabolism	209
path:ko00944	Flavone and flavonol biosynthesis	1
path:ko05219	Bladder cancer	3
path:ko00190	Oxidative phosphorylation	723
path:ko05211	Renal cell carcinoma	13
path:ko04971	Gastric acid secretion	1
path:ko00061	Fatty acid biosynthesis	184

Table S2 (continued) KEGG pathways statistics

Pathway	Pathway Definition	Number of Sequences
path:ko04115	p53 signaling pathway	4
path:ko00330	Arginine and proline metabolism	483
path:ko00270	Cysteine and methionine metabolism	365
path:ko00100	Steroid biosynthesis	1
path:ko00020	Citrate cycle (TCA cycle)	453
path:ko00901	Indole alkaloid biosynthesis	2
path:ko03460	Fanconi anemia pathway	2
path:ko05134	Legionellosis	84
path:ko01220	Degradation of aromatic compounds	52
path:ko00621	Dioxin degradation	1
path:ko05032	Morphine addiction	2
path:ko04151	PI3K-Akt signaling pathway	35
path:ko00350	Tyrosine metabolism	140
path:ko04080	Neuroactive ligand-receptor interaction	1
path:ko00630	Glyoxylate and dicarboxylate metabolism	390
path:ko00232	Caffeine metabolism	1
path:ko05016	Huntington's disease	45
path:ko02010	ABC transporters	480
path:ko03320	PPAR signaling pathway	98
path:ko00720	Carbon fixation pathways in prokaryotes	705
path:ko00920	Sulfur metabolism	129
path:ko00360	Phenylalanine metabolism	189
path:ko04978	Mineral absorption	1
path:ko00363	Bisphenol degradation	15
path:ko01230	Biosynthesis of amino acids	1372
path:ko04961	Endocrine and other factor-regulated calcium reabsorption	1
path:ko04066	HIF-1 signaling pathway	44
path:ko00120	Primary bile acid biosynthesis	6
path:ko04122	Sulfur relay system	100
path:ko00230	Purine metabolism	963
path:ko00980	Metabolism of xenobiotics by cytochrome P450	33
path:ko00592	alpha-Linolenic acid metabolism	7
path:ko01053	Biosynthesis of siderophore group nonribosomal peptides	9
path:ko05143	African trypanosomiasis	16
path:ko00521	Streptomycin biosynthesis	55

Table S3 MG-degrading characteristics comparison of Mgv-rPOD, Mgv-rLACC and Mgv-rCYP

	Mgv-rPOD	Mgv-rLACC	Mgv-rP450
Range of pH	7.0-11.0	3.0-4.0	6.0-7.0
Range of temperature (°C)	10-30	40-60	30-40
Highest degradable concentration (mg/L)	300	20	30
Decolorization efficiency (%) at highest MG concentration	97.3	63.7	36.2
Decolorization time (h)	0.67	24	24
Co-factors	H_2O_2	Cu^{2+}	NADPH
Inhibitors	Cu^{2+}, SDS	EDTA, NaCl, SDS	Cu^{2+}, Ni^{2+}, SDS

Table S4 Putative MDGs detected in this study

Classification	Annotation Result	Gene Name	Identity (%)	Complete Gene or not
laccase	*laccase**	mgv-laccase	59	Not complete
	laccase	mgv-laccase-2	61	Not complete
peroxidase	*cytochrome C peroxidase*	mgv-peroxidase-2	59	Not complete
	glutathione peroxidase	mgv-peroxidase-3	72	Not complete
	thiol peroxidase	mgv-peroxidase-4	78	Not complete
	thiol peroxidase	mgv-peroxidase-5	67	Complete
	glutathione peroxidase	mgv-peroxidase-6	73	Not complete
	thiol peroxidase, Bcp-type	mgv-peroxidase-7	61	Complete
	glutathione amide-dependent peroxidase	mgv-peroxidase-8	75	Not complete
	glutathione peroxidase	mgv-peroxidase-9	70	Not complete
	lipid hydroperoxide peroxidase	mgv-peroxidase-10	70	Not complete
	glutathione amide-dependent peroxidase	mgv-peroxidase-11	63	Not complete
	cytochrome C peroxidase	mgv-peroxidase-12	66	Complete
	cytochrome C peroxidase	mgv-peroxidase-13	66	Complete
	peroxidase	mgv-peroxidase-14	83	Not complete
	*peroxidase**	mgv-peroxidase	56	Complete
	thiol peroxidase	mgv-peroxidase-15	70	Not complete
	glutathione amide-dependent peroxidase	mgv-peroxidase-16	74	Not complete
	magnetococcus thiol peroxidase	mgv-peroxidase-17	52	Not complete
	peroxidase	mgv-peroxidase-18	90	Not complete
	glutathione peroxidase	mgv-peroxidase-19	58	Not complete
	peroxidase Tpx family thiol peroxidase	mgv-peroxidase-20	57	Not complete
	glutathione peroxidase	mgv-peroxidase-21	78	Not complete
	thiol peroxidase	mgv-peroxidase-22	77	Complete
	hydrogen peroxidase	mgv-peroxidase-23	?	Not complete
	catalase-peroxidase	mgv-peroxidase-24	74	Not complete
	peroxidase	mgv-peroxidase-25	73	Not complete
	hydroperoxidase	mgv-peroxidase-26	81	Not complete
	peroxidase	mgv-peroxidase-27	79	Not complete
	peroxidase	mgv-peroxidase-28	87	Not complete
	peroxidase	mgv-peroxidase-29	45	Not complete
	hydroperoxidase	mgv-peroxidase-30	94	Not complete
	glutathione peroxidase	mgv-peroxidase-31	71	Not complete
	peroxidase	mgv-peroxidase-32	91	Not complete

Table S4 (continued) Information of putative MDGs detected in this study

Classification	Gene Name	Position	Best Identity (%)	Complete Gene or not
peroxidase	*glutathione peroxidase*	mgv-peroxidase-33	46	Not complete
	catalase-peroxidase	mgv-peroxidase-34	60	Not complete
	catalase/hydroperoxidase HPI(I)	mgv-peroxidase-35	87	Not complete
	glutathione peroxidase	mgv-peroxidase-36	65	Not complete
	peroxidase	mgv-peroxidase-37	59	Not complete
	glutathione peroxidase	mgv-peroxidase-38	61	Not complete
	peroxidase	mgv-peroxidase-39	59	Not complete
	thiol peroxidase	mgv-peroxidase-40	69	Complete
cytochrome P450	*cytochrome P450*	mgv-p450-2	50	Not complete
	*cytochrome P450**	mgv-p450	53	Not complete

Note: gene names with * were the three MDGs chosen to verify MG-degrading activity through prokaryotic expression.

Table S5 Bacterial origin of putative MDGs in this study

Genus	Phylum	Target substance	References*
Acidovorax	*Proteobacteria*	PAHs<br>Chlorobenzenes<br>PCB<br>Nitrobenzene<br>MG	Singleton et al. 2009<br>Monferrán et al. 2005<br>Ohtsubo et al. 2012<br>Zhao and Ward 1999<br>This study
Chromobacterium:	*Proteobacteria*	Phenol<br>MG	Perpetuo et al. 2009<br>This study
Shewanella	*Proteobacteria*	Methyl Violet B<br>Naphthylaminesulfonic azo dye<br>Naphthol Green B dye<br>MG	Chen et al. 2010<br>Hong et al. 2007<br>Xiao et al. 2012<br>This study
Oscillatoria	*Cyanobacteria*	Crude Oil<br>MG	Raghukumar et al. 2001<br>Abed and Köster 2005<br>This study
Thiocapsa	*Proteobacteria*	PAHs<br>MG	Fülöp et al. 2012<br>This study
Frankia	*Actinobacteria*	Atrazine<br>MG	Rehan et al. 2014<br>This study
Nitrosomonas	*Thaumarchaeota*	Halogenated Aliphatic<br>Trichloroethylene<br>PAHs<br>MG	Vannelli et al. 1990<br>Hyman et al. 1995<br>Chang et al. 2002<br>This study
Sedimenticola:	*Proteobacteria*	Homocyclic Aromatic Compounds<br>MG	Boll et al. 2014<br>This study
Formosa	*Bacteroidetes*	Textile Industry<br>MG	Ali et al. 2011<br>This study
Spirochaeta	*Spirochaetes*	Oil<br>MG	Magot et al. 1997<br>This study
Rhodanobacter	*Proteobacteria*	lindane<br>Benzo [a] Pyrene<br>Aromatic Hydrocarbons<br>MG	Nalin et al. 1999<br>Kanaly et al. 2002<br>Arulazhagan et al. 2014<br>Bacosa et al. 2012<br>This study
Salinibacter	*Bacteroidetes*	Petroleum Hydrocarbons<br>MG	Martins and Peixoto 2012<br>This study
Desulfuromonas	*Proteobacteria*	MG	This study
Geopsychrobacter	*Proteobacteria*	MG	This study
Cellvibrio	*Proteobacteria*	MG	This study
Simiduia	*Proteobacteria*	MG	This study
Nitrosomonas	*Proteobacteria*	MG	This study
Leucothrix	*Proteobacteria;*	MG	This study

Magnetococcus	*Proteobacteria;*	MG	This study
Gemmobacter	*Proteobacteria*	MG	This study
Kangiella	*Proteobacteria*	MG	This study
Zooshikella	*Proteobacteria*	MG	This study
Marinobacter	*Proteobacteria*	MG	This study
Draconibacterium	*Bacteroidetes*	MG	This study
Bizionia	*Bacteroidetes*	MG	This study
Gaetbulibacter	*Bacteroidetes*	MG	This study
Mariniradius	*Bacteroidetes*	MG	This study
Imtechella	*Bacteroidetes*	MG	This study
Gelidibacter	*Bacteroidetes*	MG	This study

* showed that partial putative MDGs detected in this study were reported in previous references.

1.2 Supplementary Figures

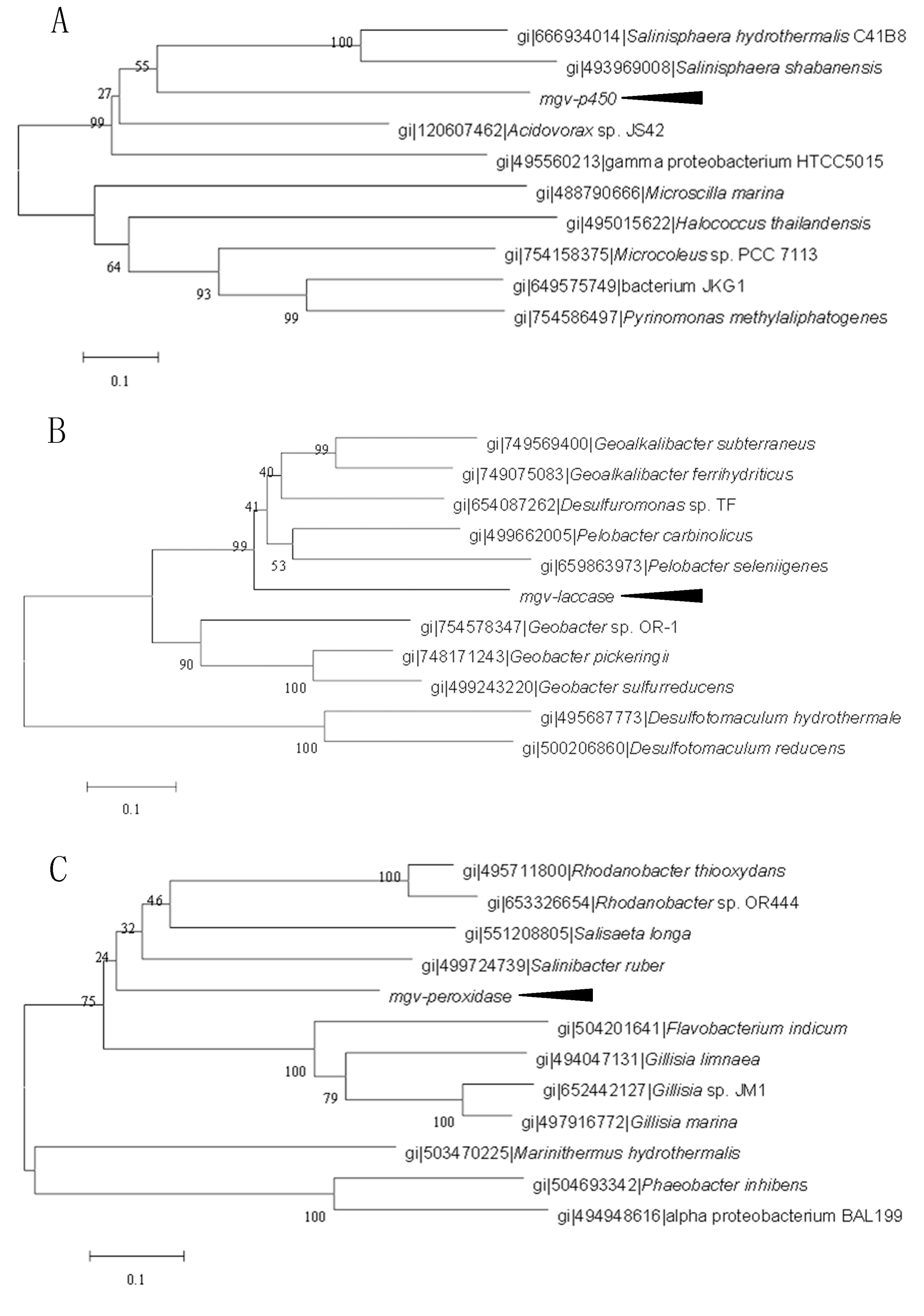

Fig. S1 Phylogenetic tree (neighbor-joining method) of *mgv-laccase* (A), *mgv-450* (B) and *mgv-peroxidase* (C). Bootstrap values of 500, calculated from 1,000 bootstrap trees are indicated at the nodes. The numbers in the brackets are the GenBank accession numbers of the referenced amino acid sequences. The scale bar represents 0.1 amino acid substitution per position

A

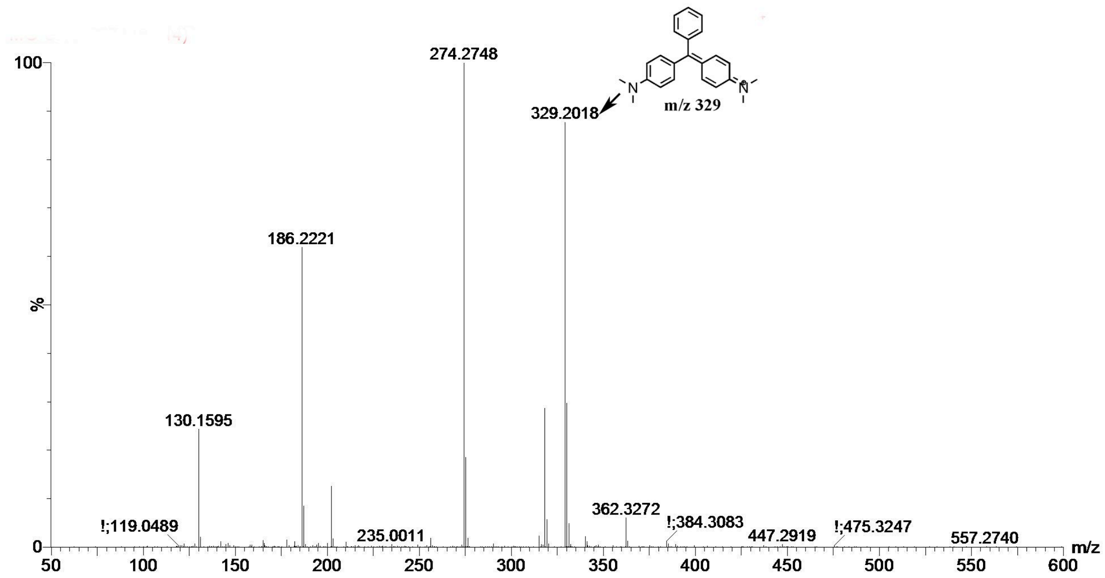
100
%
0
274.2748
329.2018
m/z 329
186.2221
130.1595
!;119.0489
235.0011
362.3272
!;384.3083
447.2919
!;475.3247
557.2740
m/z
50
100
150
200
250
300
350
400
450
500
550
600

B

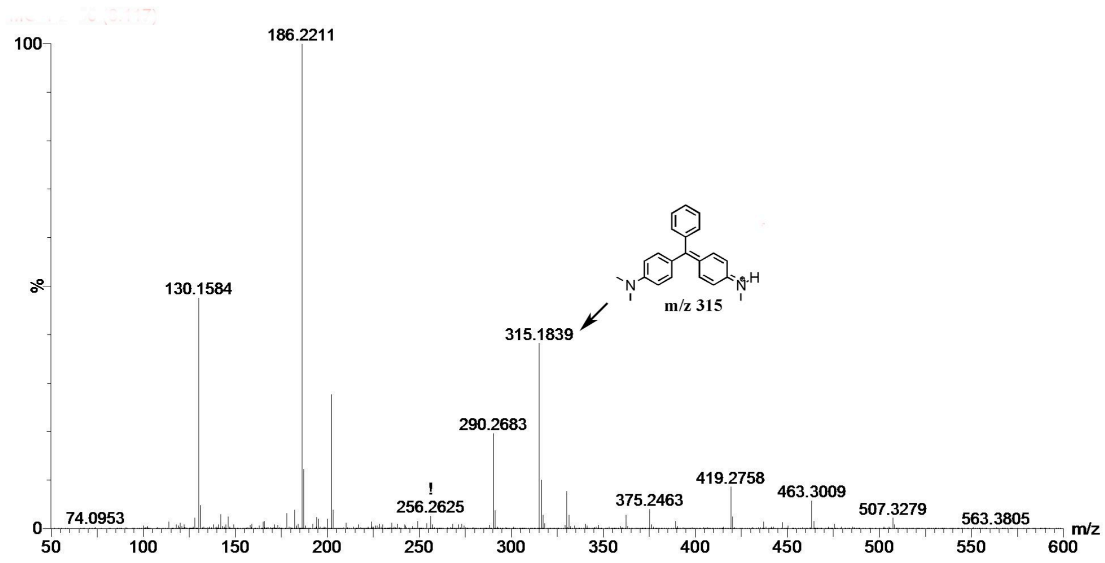
100
%
0
186.2211
130.1584
m/z 315
315.1839
290.2683
!
256.2625
74.0953
375.2463
419.2758
463.3009
507.3279
563.3805
m/z
50
100
150
200
250
300
350
400
450
500
550
600

C

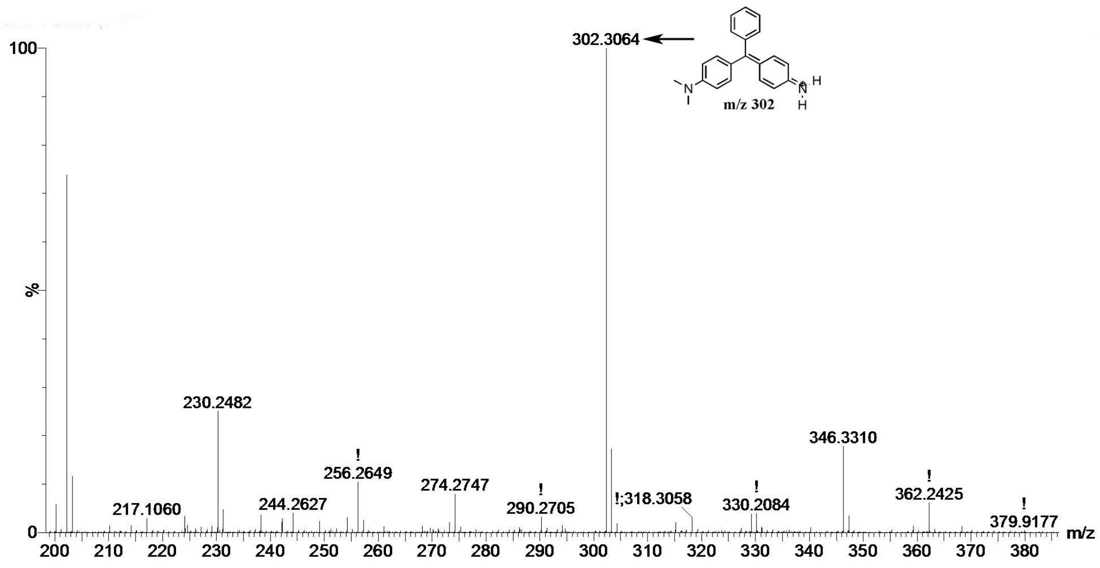

302.3064
m/z 302
100
%
0
230.2482
217.1060
244.2627
256.2649
274.2747
290.2705
!;318.3058
330.2084
346.3310
362.2425
379.9177
m/z
200 210 220 230 240 250 260 270 280 290 300 310 320 330 340 350 360 370 380

D

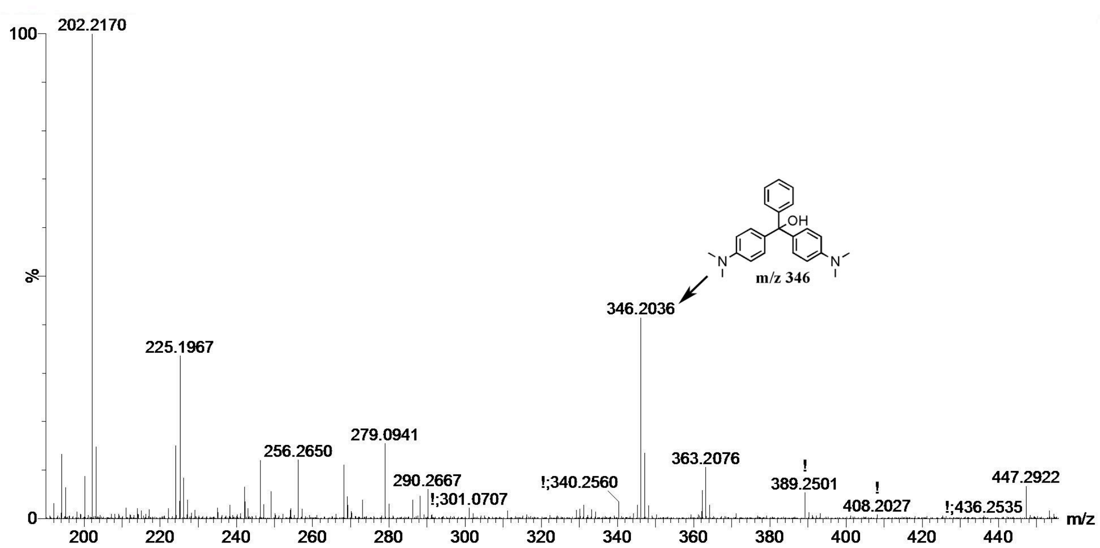

202.2170
100
%
0
m/z 346
346.2036
225.1967
256.2650
279.0941
290.2667
!;301.0707
!;340.2560
363.2076
389.2501
408.2027
!;436.2535
447.2922
m/z
200 220 240 260 280 300 320 340 360 380 400 420 440

E

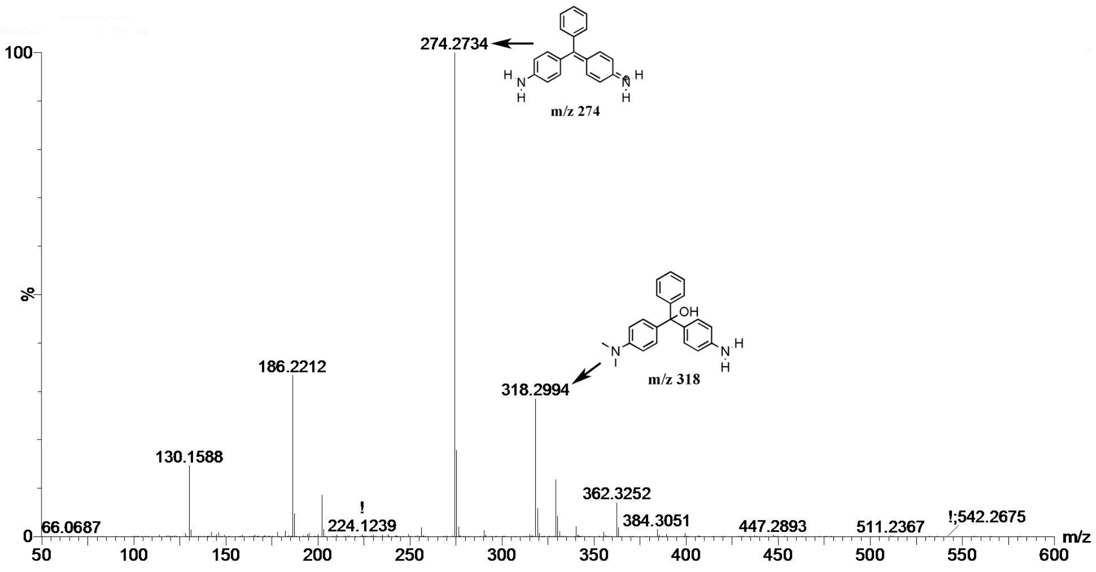
274.2734
m/z 274
100
%
0
186.2212
130.1588
66.0687
224.1239
318.2994
m/z 318
OH
362.3252
384.3051
447.2893
511.2367
542.2675
m/z
50
100
150
200
250
300
350
400
450
500
550
600

F

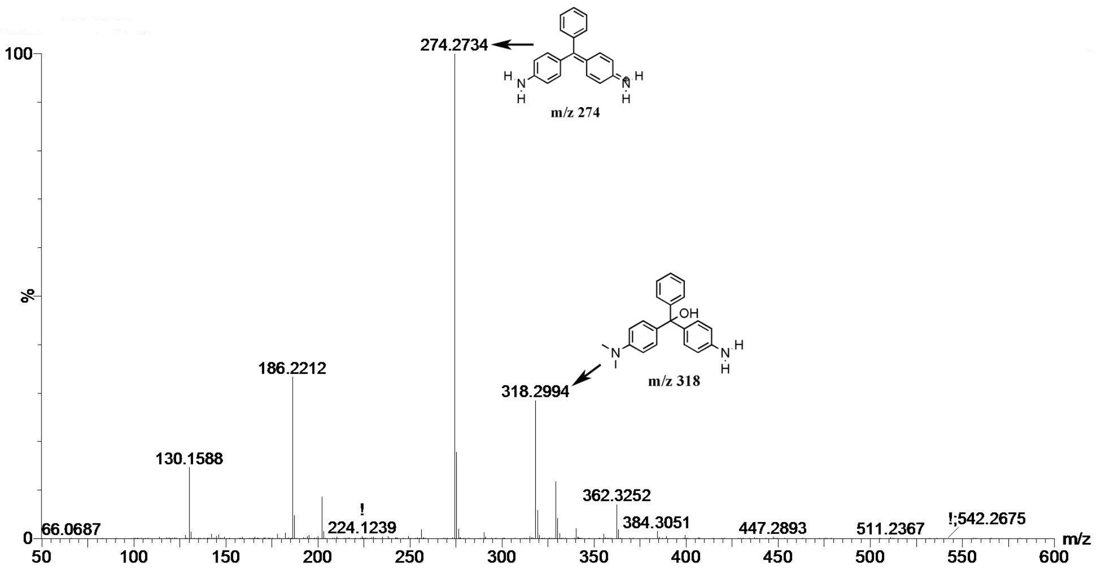
274.2734
m/z 274
100
%
0
186.2212
130.1588
66.0687
224.1239
318.2994
m/z 318
OH
362.3252
384.3051
447.2893
511.2367
542.2675
m/z
50
100
150
200
250
300
350
400
450
500
550
600

G

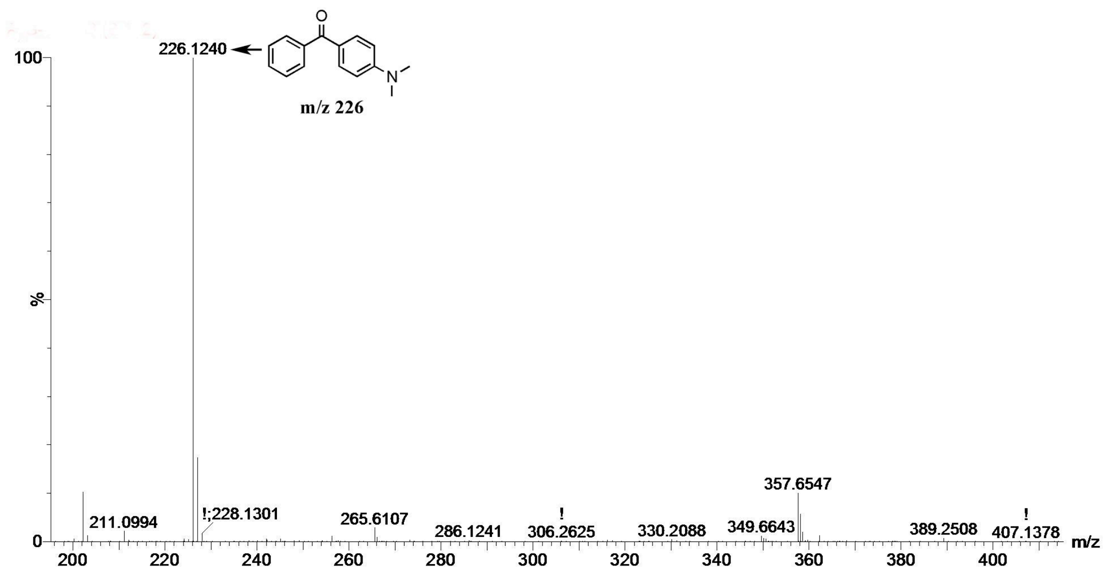
226.1240
m/z 226
100
%
0
211.0994
!;228.1301
265.6107
286.1241
!
306.2625
330.2088
349.6643
357.6547
389.2508
!
407.1378
m/z
200
220
240
260
280
300
320
340
360
380
400

H

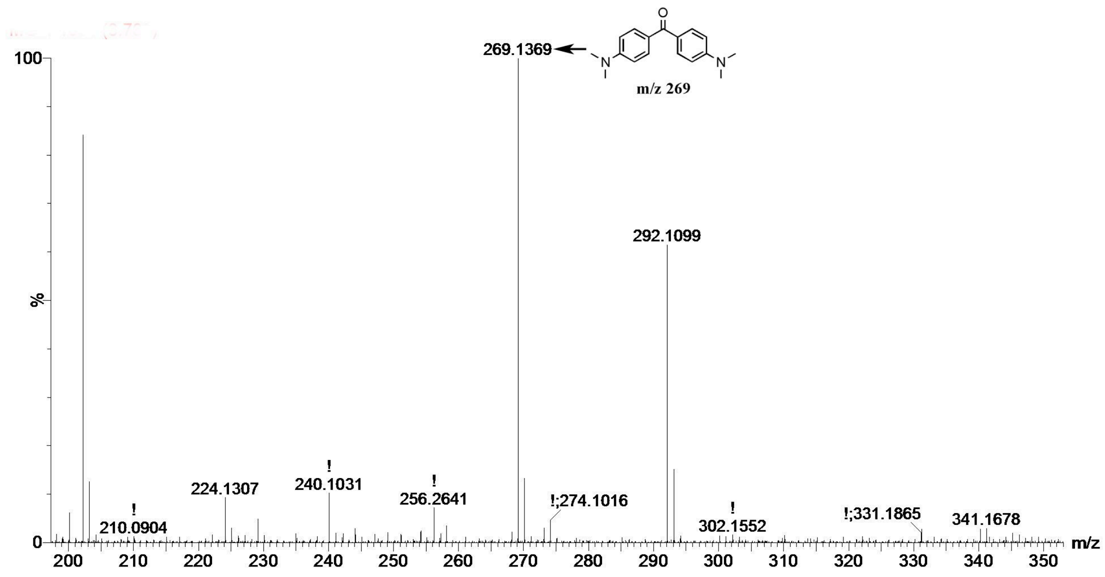
269.1369
m/z 269
100
%
0
292.1099
!
210.0904
224.1307
!
240.1031
!
256.2641
!;274.1016
!
302.1552
!;331.1865
341.1678
m/z
200
210
220
230
240
250
260
270
280
290
300
310
320
330
340
350

I

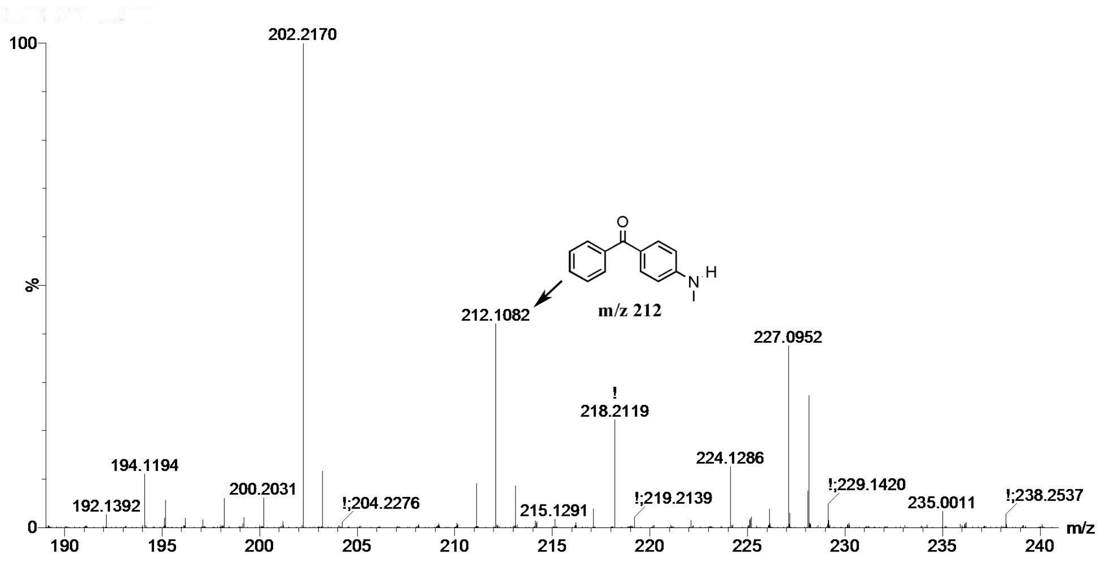

202.2170
212.1082
m/z 212
227.0952
!
218.2119
224.1286
194.1194
200.2031
192.1392
!;204.2276
215.1291
!;219.2139
!;229.1420
235.0011
!;238.2537
100
%
0
190
195
200
205
210
215
220
225
230
235
240
m/z

J

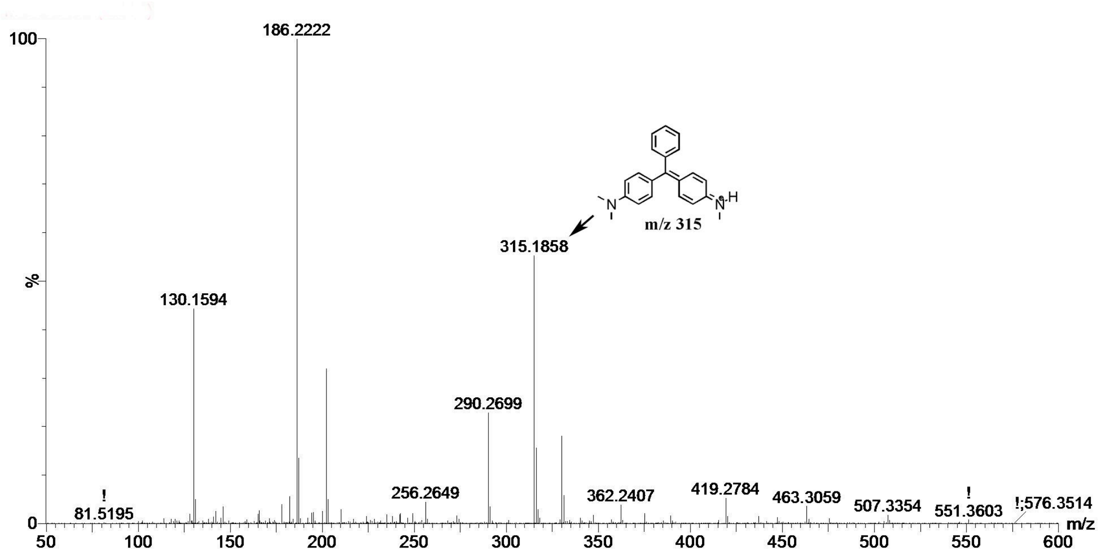

186.2222
m/z 315
315.1858
130.1594
290.2699
!
81.5195
256.2649
362.2407
419.2784
463.3059
507.3354
!
551.3603
!;576.3514
100
%
0
50
100
150
200
250
300
350
400
450
500
550
600
m/z

K

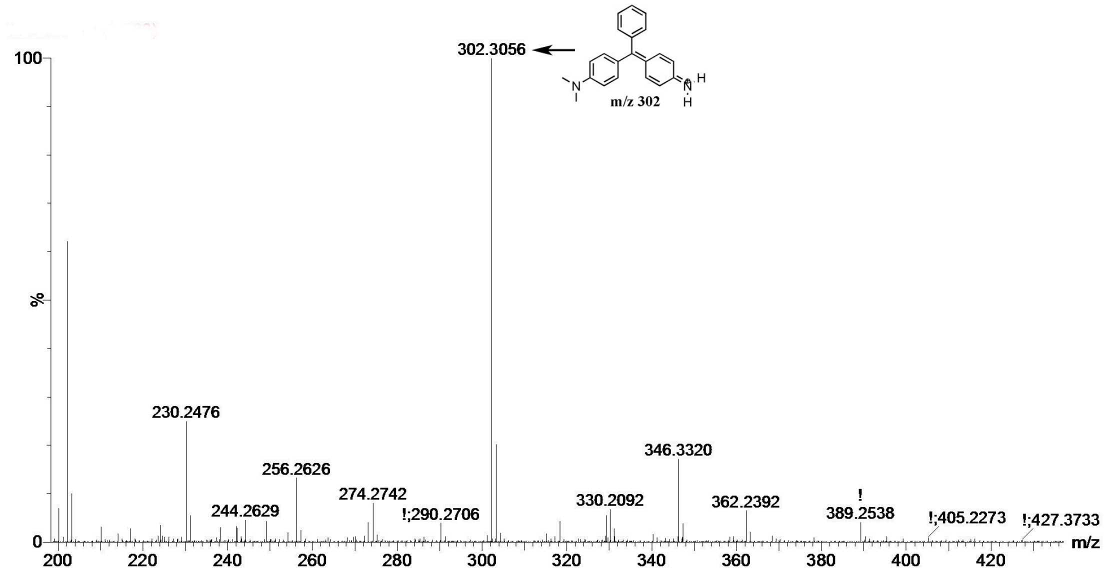

302.3056
m/z 302
100
%
0
230.2476
244.2629
256.2626
274.2742
!;290.2706
330.2092
346.3320
362.2392
!
389.2538
!;405.2273
!;427.3733
m/z
200
220
240
260
280
300
320
340
360
380
400
420

L

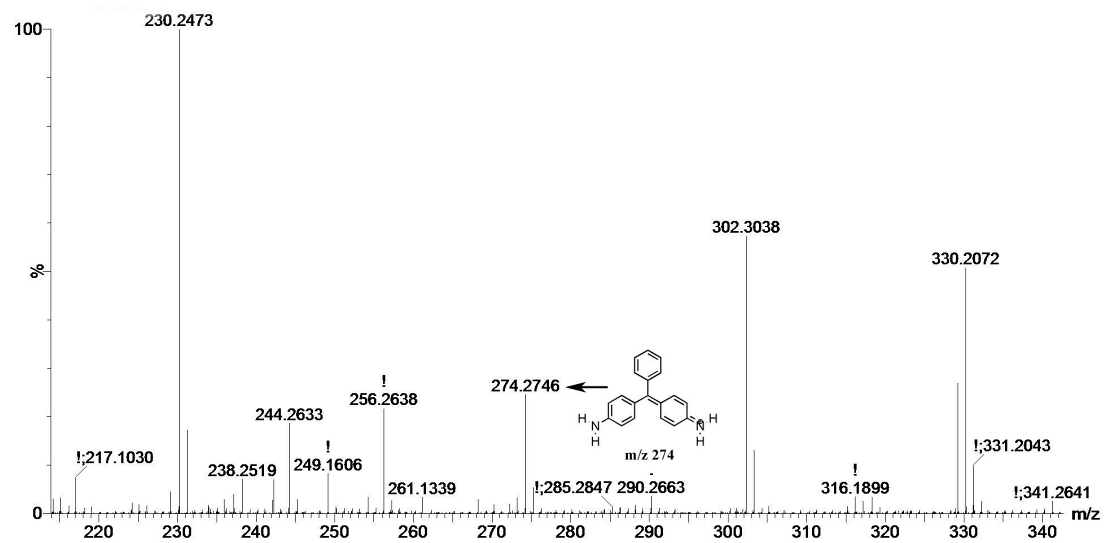

100
230.2473
%
0
302.3038
330.2072
274.2746
m/z 274
!
256.2638
244.2633
!
249.1606
!;217.1030
238.2519
261.1339
!;285.2847
290.2663
!
316.1899
!;331.2043
!;341.2641
m/z
220
230
240
250
260
270
280
290
300
310
320
330
340

M

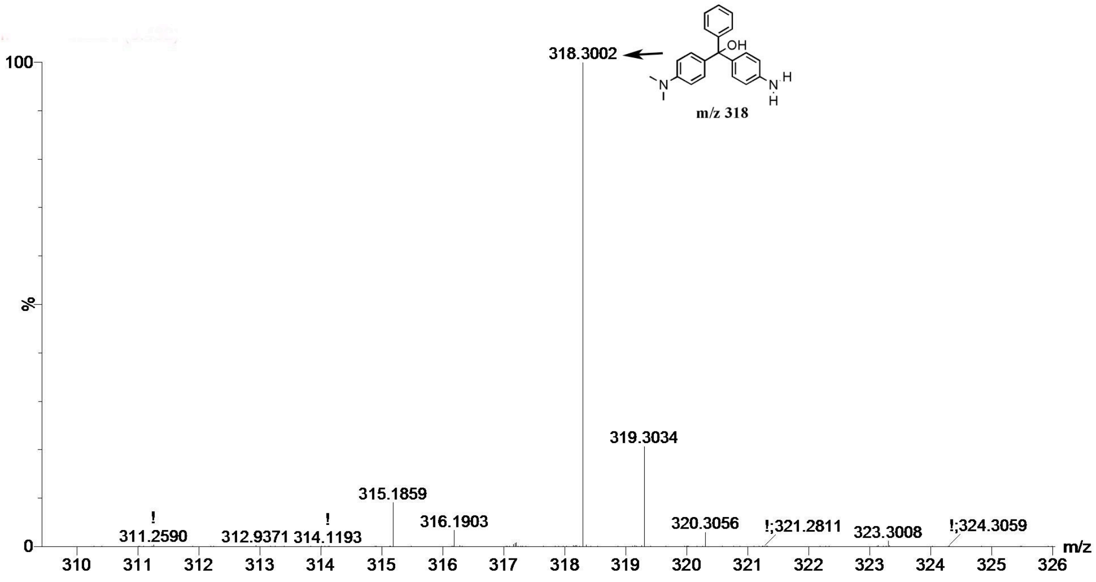

N

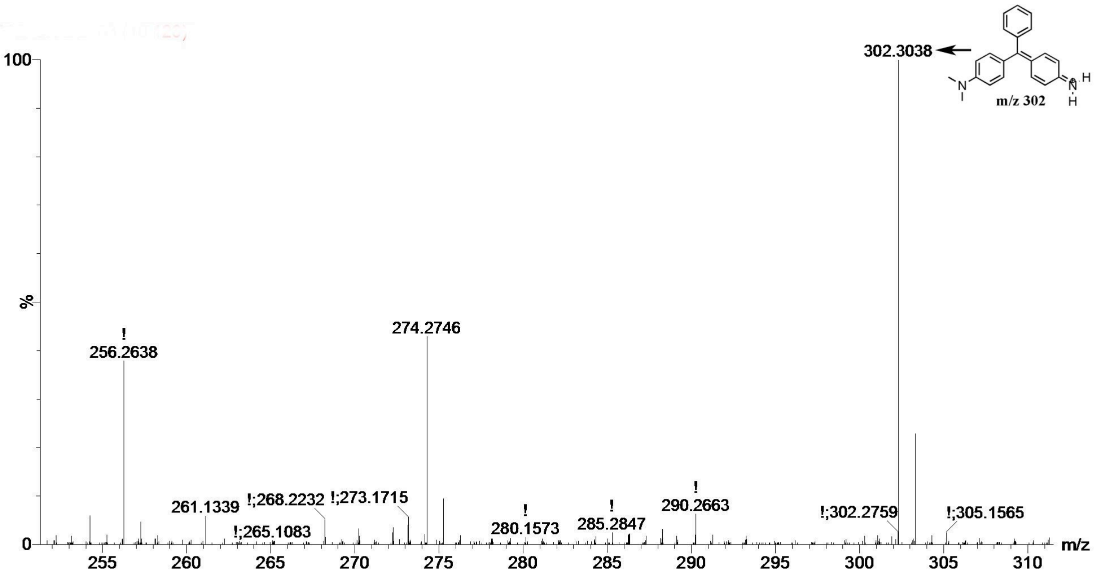

Fig. S2 The intermediates of MG degradation by Mgv-rPOD (B-I), Mgv-rLACC (J-L) and Mgv-rCYP (M, N). (A) MG (m/z 329); (B, J) desmethyl-MG (m/z 315); (C, K, N) didesmethyl-MG (m/z 302); (D) hydroxyl-MG (m/z 346); (E, M) hydroxyl(didesmethyl)-MG (m/z 318); (F, L) tetradesmethyl-LMG (m/z 274); (G) 4-(dimethylamino) benzophenone (m/z 226); (H) Michler’s ketone (m/z 269); (I) 4-(methylamino) benzophenone (m/z 212).

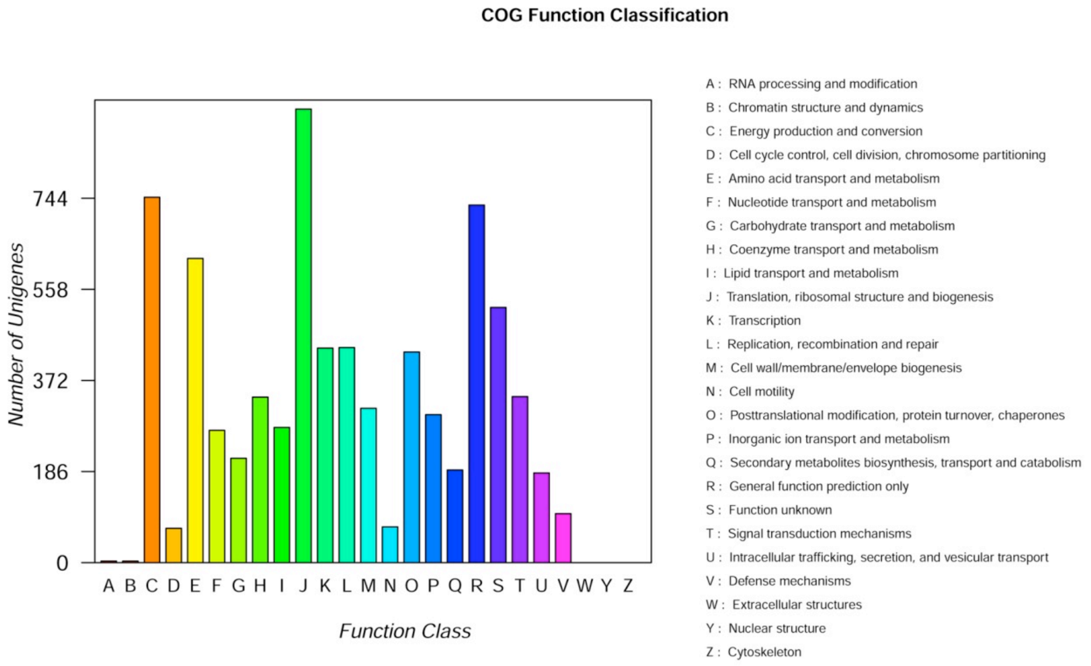

Fig. S3 The function class analysis of the genes from the sequencing data in this study.
